# Supplementary material for: Trimethylamine N-Oxide Exacerbates Neuroinflammation and Motor Dysfunction in an Acute MPTP Mice Model of Parkinson’s Disease
Source: Brain Sci. 2023 May 12;13(5):790. doi: 10.3390/brainsci13050790 (PMC10216692; doi:10.3390/brainsci13050790)
Supplement: Supplementary file 1 [file brainsci-13-00790-s001.zip › brainsci-2348717-supplementary.pdf]

Supplementary materials:

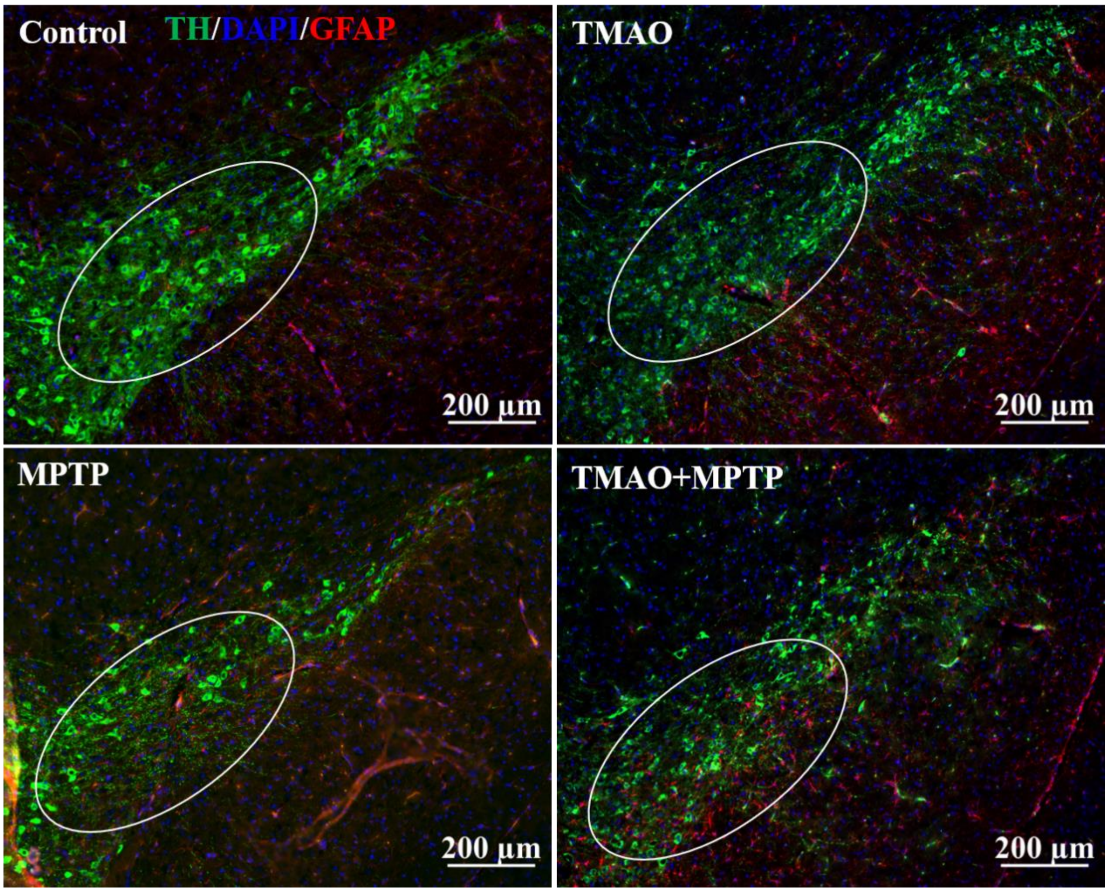

Figure S1: Double immunofluorescence staining images for TH and GFAP in the substantia nigra. Representative photomicrographs of TH (dopaminergic neuron marker, green) and GFAP (astrocytes marker, red) staining in the substantia nigra. The scale bar is 200 μm.

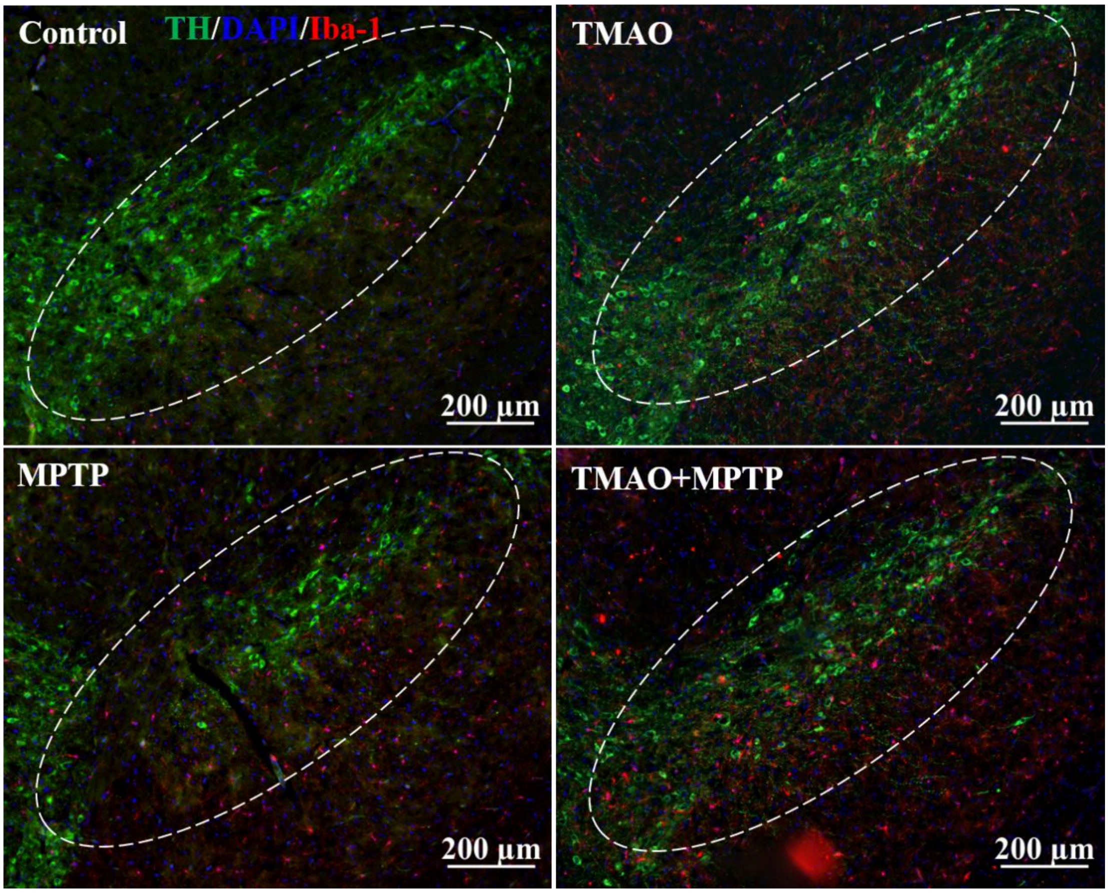

Figure S2: Double immunofluorescence staining images for TH and Iba-1 in the substantia nigra. Representative photomicrographs of TH (dopaminergic neuron marker, green) and Iba-1 (microglia marker, red) staining in the substantia nigra. The scale bar is 200 μm.
